# Photostable Red-Emitting Fluorescent Rhein-Magnesium(Ⅱ) Coordination Polymer Nanodot-Based Nanostructures With a Large Stokes Shift for Imaging Mitochondria in Cancer Cell

**DOI:** 10.3389/fonc.2021.758268

**Published:** 2021-10-25

**Authors:** Qin Jiang, Ke Du, Yuhang Jiang, Yuhan Liu, Chen Han, Zhihui Yin, Ying Wang, Xiaoyan Gao

**Affiliations:** School of Chinese Materia Medica, Beijing University of Chinese Medicine, Beijing, China

**Keywords:** nanomaterials, cancer cell, mitochondria, fluorescence imaging, red emission

## Abstract

The mitochondria play a significant role in many cellular processes and are recognized as one of the most important therapeutic targets in cancer. Direct long-term imaging of the mitochondria is very crucial for treating cancer. However, the development of a red-emitting mitochondrial probe with a large Stokes shift and photostability remains highly challenging. Fluorescent metal complexes with superior physicochemical property have emerged as new fluorescent nanomaterials due to their increasing advantages in bioimaging. Herein, a luminescent pitaya-type nanostructure based on rhein-magnesium(II) (Rh-Mg) coordination polymer nanodots was used as a fluorescent nanoprobe to selectively image the mitochondria benefiting from the introduction of triphenylphosphine. The as-prepared Rh-Mg nanodot-based nanoprobe showed red emission peaking at 620 nm, a large Stokes shift (100 nm), and excellent photostability as compared with commercial mitochondrial probes. Due to these extraordinary features, this fluorescent nanoprobe was successfully used for mitochondrial targeting imaging of live cancer cell line Neuro-2a (mouse neuroblastoma) and BV2 microglial cells. Therefore, our results pave a new way for the design of fluorescent nanoprobes for imaging mitochondria in cancer cell.

## Introduction

Among all cellular organelles, the mitochondria are known as the “power house” of mammalian cells and play crucial roles in biosynthesis, intracellular signal transduction, energy homeostasis, and apoptosis regulation (1–4). Increasing evidence suggests that mitochondrial dysfunction is a key hallmark of many cancers, as reduced mitochondrial respiration has been observed in cancer cells, a phenomenon referred to as “Warburg effect” (5–7). Therefore, as an important therapeutic target in cancer, it is significant to track and image the mitochondria.

Fluorescence imaging (8–12), as a crucial diagnostic method, has advantages in mitochondrial imaging because of its good sensitivity, high spatial and temporal resolution, and easy operation (13–15). However, most conventional fluorescent dyes, especially commercialized probes, such as MitoTracker Green and rhodamine 123, fail to provide long-period tracking due to severe photobleaching. With low cytotoxicity and high stability, carbon dots (CDs) have been designed for organelle imaging, such as the nucleus and lysosomes (16). So far, only very few examples of CD-based nanostructures have been proposed as fluorescent nanoprobes for selective mitochondrial targeting, but they usually only have the emission in the blue light range, which greatly limits their application (17, 18). Recently, although one case of CDs can be emitted in the red light region, its Stokes shift is very small, only about 20 nm (19). Small Stokes shift will decrease the detection sensitivity of probes in practical application. Thus, the development of a red light-emitting mitochondrial probe with a large Stokes shift and photostability is imperative but exceedingly challenging.

Very recently, fluorescent metal complexes have moved into the spotlight as mitochondrial probes due to their superior physicochemical property, high photostability, and large Stokes shifts (20–25). In particular, transition metal complexes have been developed as excellent candidate. For example, a series of fluorescent ruthenium(II)-, iridium(III)-, and rhodium(III)-based BODIPY complexes were specifically localized in the mitochondria of live cell and succeeded in live cell imaging (26). However, precious metal complexes like ruthenium(II), iridium(III), and rhodium(III) suffer from limited resources and high cost that limit their applications in a large scale.

Inspired by the coordination of rhein and metal ions, we selected the inexpensive magnesium ion among masses of metal ions to develop a rhein-magnesium(II) (Rh-Mg) coordination polymer nanodots and functionalized them with triphenylphosphine (Rh-Mg-PVP@SiO_2_-TPP NPs) to target the mitochondria in cancer cells. The as-prepared Rh-Mg-PVP@SiO_2_-TPP NPs exhibited good photostability, favorable biocompatibility, and superior fluorescence properties like red emission with a large Stokes shift. *In vitro*, Rh-Mg-PVP@SiO_2_-TPP NPs were mainly concentrated in the mitochondria and successfully used for mitochondrial targeting imaging of Neuro-2a cells, an aggressive mouse neuroblastoma cell line, and BV2 cells. Moreover, the Rh-Mg-PVP@SiO_2_-TPP NPs were suitable for long-term cell imaging. Thus, the as-prepared Rh-Mg-PVP@SiO_2_-TPP NPs may represent a promising candidate probe to image the mitochondria and provide important information about cancer presence and progression.

## Materials and Methods

### Chemicals

The chemicals used in the study were as follows: rhein (Aladdin), magnesium chloride hexahydrate (Aladdin), polyvinylpyrrolidone (PVP, Aladdin, average molecular weight 8,000, K16-18), 1-ethyl-(3-dimethylaminopropyl) carbon diimine hydrochloride (EDC·HCl, Aladdin), (4-carboxybutyl) triphenyl phosphine bromide (TPP-COOH, Aladdin), N-hydroxysuccinimide (NHS, Aladdin), 3-aminopropyl triethoxysilane (APTES, Aladdin), tetraethyl orthosilicate (TEOS, Sigma), and ammonia (25%–28%, Rohn’s reagent).

Deionized (DI) water (18.2 MΩ cm, Millipore) was used for the preparation of solutions. All chemicals were utilized as received without further purification or processing.

### Instrumentation

The transmission electron microscope images were obtained using the JEM-2100 (JEOL, Japan) transmission electron microscope operating at an accelerated voltage of 200 kV. A ZEN 3600 instrument (Malvern, UK) was used to measure dynamic light scattering and zeta potential. Fourier transform infrared spectra (FT-IR) were measured on a Nicolet™ iS™ 10 spectrophotometer (Thermo Fisher Scientific, USA) at 4 cm^−1^ resolution with 32 scans. UV–vis absorbance spectra were recorded using a UH5300 spectrophotometer (Hitachi High-Technologies Corporation, Shanghai). Fluorescence spectroscopy was recorded using a PerkinElmer LS45 (PerkinElmer, USA). The images of confocal scanning laser microscopy were taken using a TCS SP8 microscope (Leica, Germany). The images of the inverted fluorescence microscope were captured by ECLIPSE-Ts2 microscope (Nikon Instruments Inc., Melville, NY, USA). *In/ex vivo* fluorescence images were taken by MIIS Small Animal Imaging System (Molecular Devices, USA).

### Synthetic Procedures

#### Synthesis of Rh-Mg-PVP

Rhein (0.9 mg) and MgCl_2_ (0.264 mg) were dissolved in 21 ml methanol and 0.12 ml ammonia (25%–28%) and then stirred for 30 min at room temperature. PVP (2.4 mg) was added for further stirring for 30 min to obtain the Rh-Mg-PVP complex solution.

#### Synthesis of Rh-Mg-PVP@SiO_2_-TPP NPs

In order for the nanomaterial to have the targeting property, the surface of the Rh-Mg-PVP complex was firstly coated with silica shell layer; 10.15 ml methanol and 2.85 ml ammonia (25%–28%) were added to 7 ml Rh-Mg-PVP complex solution, and magnetic stirring was done at room temperature for 30 min for even dispersion. Then, 80 μl TEOS was added and the solution was stirred for 30 min after each addition of TEOS and stirred overnight after adding all to obtain Rh-Mg-PVP@SiO_2_.

The surface of Rh-Mg-PVP@SiO_2_ first underwent amination with APTES. Forty milliliters of Rh-Mg-PVP@SiO_2_ NP solution was added to a 100-ml round bottom flask, then about 30 μl of APTES was added. The mixture was stirred well and refluxed for 1 h in a 65°C condensation reflux device. The condensing tube was removed and then transferred to a water bath at 30°C and stirred for 12 h. TPP-COOH (1 equivalent) was dissolved in ethanol (1 ml); then, EDC (5 equivalent) and NHS (5 equivalent) were added. The active carboxylate groups are obtained by stirring for 2 h. Finally, NPs-NH_2_ was conjugated through the condensation reaction between the −NH_2_ group and activated −COOH group (TPP-COOH). Then, the solution was centrifuged and washed with water several times. The same operation was repeated twice. The as-prepared nanocomposites were named Rh-Mg-PVP@SiO_2_-TPP NPs.

### Density Functional Theoretical Calculation

To examine the optimized structures of the compounds and its relative stability, geometry optimization with Gaussian 16 program package using standard B3LYP-6-31G (d, p) basis set was performed (27). Vibration analysis showed that the optimized structure was in accordance with the minimum points on the potential energy surface.

### Cell Culture

The mouse microglia cell line BV2 and the mouse neuroblastoma cell line Neuro-2a were purchased from the ATCC. Cells were cultured at 37°C under 5% CO_2_ in DMEM containing 10% (vol/vol) FBS (Sigma-Aldrich), 2 mM l-glutamine, 100 U/ml penicillin, and 100 μg/ml streptomycin (Gibco).

### Cytotoxicity Assay

The *in vitro* cytotoxicity of Rh-Mg-PVP@SiO_2_-TPP NPs was measured using a Cell Counting Kit-8 (CCK-8) assay in BV2 cell and Neuro-2a cell lines. Briefly, cells growing in log phase were seeded into a 96-well plate at a density of 5,000 cells per well (100 μl). The Rh-Mg-PVP@SiO_2_-TPP NPs (100 ml/well) at concentrations of 25, 50, 100, 200, and 400 μg ml^−1^ were added to the wells of the treatment group, and DMEM also added to the negative control group, respectively. The cells were incubated for 24 h at 37°C under 5% CO_2_. After incubation for 24 h, the medium was removed and 100 μl of CCK-8 solution was added. After 30 min, the absorbance at 450 nm was measured by a Multiskan MK3 microplate reader to determine the cell viabilities.

### Cell Imaging

BV2 cells and Neuro-2a cells were plated on 15 mm small confocal laser dishes and allowed to adhere for 24 h, and the cell density was 5 × 10^4^ ml^−1^ for 24 h, and the Rh-Mg-PVP@SiO_2_-TPP NP solution was prepared with 200 μg ml^−1^. The solution was incubated for 2, 4, and 6 h after administration. Then, the culture medium was removed and the cells were washed with PBS (1 ml) twice. One milliliter of MitoTracker Green working fluid was added and then put back into the incubator for incubation for 10–15 min. MitoTracker Green was sucked away, PBS was added along the wall, and the cells were gently washed three times by cross shaking. Finally, 1 ml of PBS was added and photographed under a confocal laser microscope. LysoTracker Green was used to investigate the distribution of nanomaterials in lysosomes, and the operation was similar to the above experiment. Luminescence imaging was performed with a PerkinElmer UltraVIEW VoX confocal fluorescence microscope and a 60 oil-immersion objective lens. Cells incubated with Rh-Mg-PVP@SiO_2_-TPP NPs were excited with a laser at 561 nm. MitoTracker Green and LysoTracker Green were excited with a laser at 488 nm.

### Photostability Investigation

Rh-Mg-PVP@SiO_2_-TPP NPs (200 μg ml^−1^) were co-incubated with BV2 cells for 2 h. After being washed with PBS, the commercially available MitoTracker Green solution was added and incubated for 15 min. The cells were placed under a confocal laser microscope and subjected to continuous laser irradiation at 561 nm for about 1 h. The fluorescence images of the red channel and the green channel within 1 h were collected and compared with the fluorescence intensity of the commercially available probe to investigate the photostability of Rh-Mg-PVP@SiO_2_-TPP NPs (19). ImageJ software was used to evaluate the fluorescence intensity of each image.

### Animals and Optical Imaging Experiments

Five-month-old male ICR (Institute of Cancer Research) mice weighing 20–30 g were provided by SiPeiFu (Beijing, China) and raised in the Animal Experimental Center of Beijing University of Chinese Medicine. Animal experiments were approved by the Animal Care and Ethics Committee of Beijing University of Chinese Medicine.

For the *in vivo* fluorescence imaging, ICR mice were first anesthetized intraperitoneally by 10% chloral hydrate (0.1 ml/10 g animal weight). Then, mice were subcutaneously injected with 6.848 mg/kg of Rh-Mg-PVP@SiO_2_-TPP NPs in the left posterior thigh, and fluorescence imaging was performed before and after injection. For *ex vivo* organ imaging, mice were firstly injected with 6.848 mg/kg of Rh-Mg-PVP@SiO_2_-TPP NPs through the tail vein. Then, at different time points postinjection (1, 4, and 12 h), mice were anesthetized, sacrificed, and dissected. The dissected organs, including the heart, liver, kidney, spleen, and lung, were removed and imaged. *In vivo* and *ex vivo* fluorescence imaging was performed with MIIS small animal imaging system (Molecular Devices, *in vivo* fluorescent imaging: excitation wavelength = 500 nm; *ex vivo* organ imaging: excitation wavelength = 560 nm). The analysis of each image was conducted by ImageJ software.

## Results and Discussion

**SCHEME 1:**
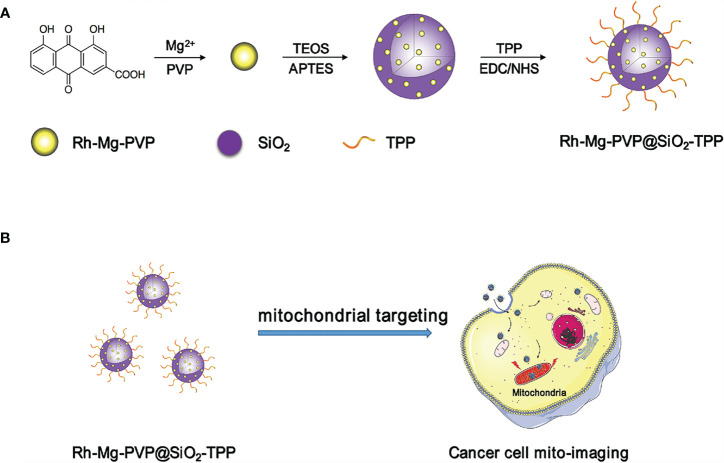
Schematic illustration of the synthesis **(A)** and application **(B)** of Rh-Mg-PVP@SiO_2_-TPP NPs.

**Figure 1:**
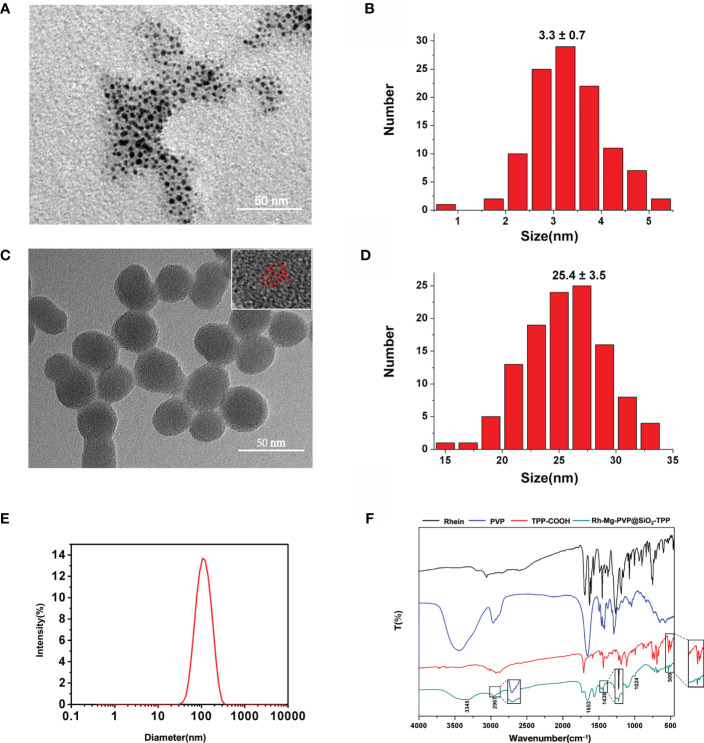
**(A)** TEM image and **(B)** size distribution of Rh-Mg coordination polymer nanodots. **(C)** TEM image and **(D)** size distribution of Rh-Mg-PVP@SiO_2_-TPP NPs. Individual Rh-Mg coordination polymer nanodots are outlined by red cycles. **(E)** Hydrodynamic size distribution of Rh-Mg-PVP@SiO_2_-TPP NPs. **(F)** FT-IR of rhein, PVP, TPP-COOH, and Rh-Mg-PVP@SiO_2_-TPP NPs.

The synthesis procedure of Rh-Mg-PVP@SiO_2_-TPP NPs is illustrated in **Scheme 1**. Firstly, rhein-magnesium (Rh-Mg) coordination polymer nanodots with an average particle size of ∼3.3 nm were first synthesized in methanol phase (**Figures 1A, B**). Second, the coordination polymer nanodots were protected with PVP(Rh-Mg-PVP) to avoid aggregation and to increase stability (28). Using a modified Stöber method (29), a SiO_2_ shell was fabricated, and multiple Rh-Mg-PVP nanodot core-silica shell nanoparticles (multi-Rh-Mg-PVP@SiO_2_ NPs) were obtained. The TEM images showed that the size of Rh-Mg-PVP@SiO_2_ was ∼30 nm in diameter with a narrow size distribution (**Supplementary Figure S1**, **Supplementary Material**). In addition, small spherical particles were observed in the TEM images dispersed in the SiO_2_ nanomaterials. The size of these spherical particles was ∼2.4 nm according to the ImageJ analysis, which was similar to the size of Rh-Mg-PVP. Thus, after coating the silica shell layer, each silica nanoparticle encapsulated several Rh-Mg-PVP nanodots, forming a similar “pitaya-type” structure. It was preliminarily inferred from the FT-IR spectra that the SiO_2_ shell was successfully coated. As can be seen from **Supplementary Figure S2**, 1,092 cm^−1^ is the absorption peak of Si–O–Si antisymmetric stretching vibration, and 799 cm^−1^ is the absorption peak of Si–O–Si symmetric stretching vibration. Accordingly, it can be concluded that silica particles with a large amount of hydroxyl groups on the surface were prepared by the classical Stöber method. To enable nanodots to target the mitochondria, the Rh-Mg-PVP@SiO_2_ first underwent amination with APTES. Then, the mitochondrial targeting group (4-carboxybutyl) triphenylphosphine bromide was activated using EDC and NHS (30, 31). Finally, Rh-Mg-PVP@SiO_2_-NH_2_ was conjugated through the condensation reaction between the −NH_2_ group and activated −COOH group, and the as-prepared nanocomposites were named Rh-Mg-PVP@SiO_2_-TPP NPs. TEM images (**Figures 1C, D** **)** showed that the prepared Rh-Mg-PVP@SiO_2_-TPP NPs were spherical with a particle size of 25.4 ± 3.5 nm. The average hydrodynamic particle size of Rh-Mg-PVP@SiO_2_-TPP NPs is about 102.7 nm, and the PDI value is 0.143, which indicated that the Rh-Mg-PVP@SiO_2_-TPP NPs have narrow particle size distributions (**Figure 1E**). In the FT-IR spectra of Rh-Mg-PVP@SiO_2_-TPP NPs (**Figure 1F**), the characteristic peak at 1,113 cm^−1^ was the stretching vibration peak of the P–C bond. The absorption peaks located at 509 and 692 cm^−1^ were attributed to the bending vibration of the P–C bond. Thus, the TPP-COOH was successfully anchored to the Rh-Mg-PVP@SiO_2_. The surface modification process was further monitored with *ζ* potential evaluations. The increase of *ζ* potentials from −27.4 to +30.1 mV proved the successful conjugation of TPP (**Supplementary Figure S3**, **Supplementary Material**).

**Figure 2:**
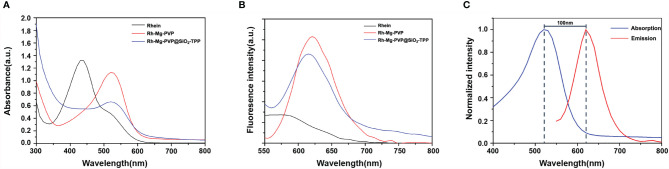
**(A)** UV–vis absorption spectra and **(B)** fluorescence emission spectra of rhein, Rh-Mg-PVP, and Rh-Mg-PVP@SiO_2_-TPP NPs. **(C)** Absorption and emission spectra (normalization) of Rh-Mg-PVP@SiO_2_-TPP NPs (*λ*_ex_ = 520 nm).

**Figure 3:**
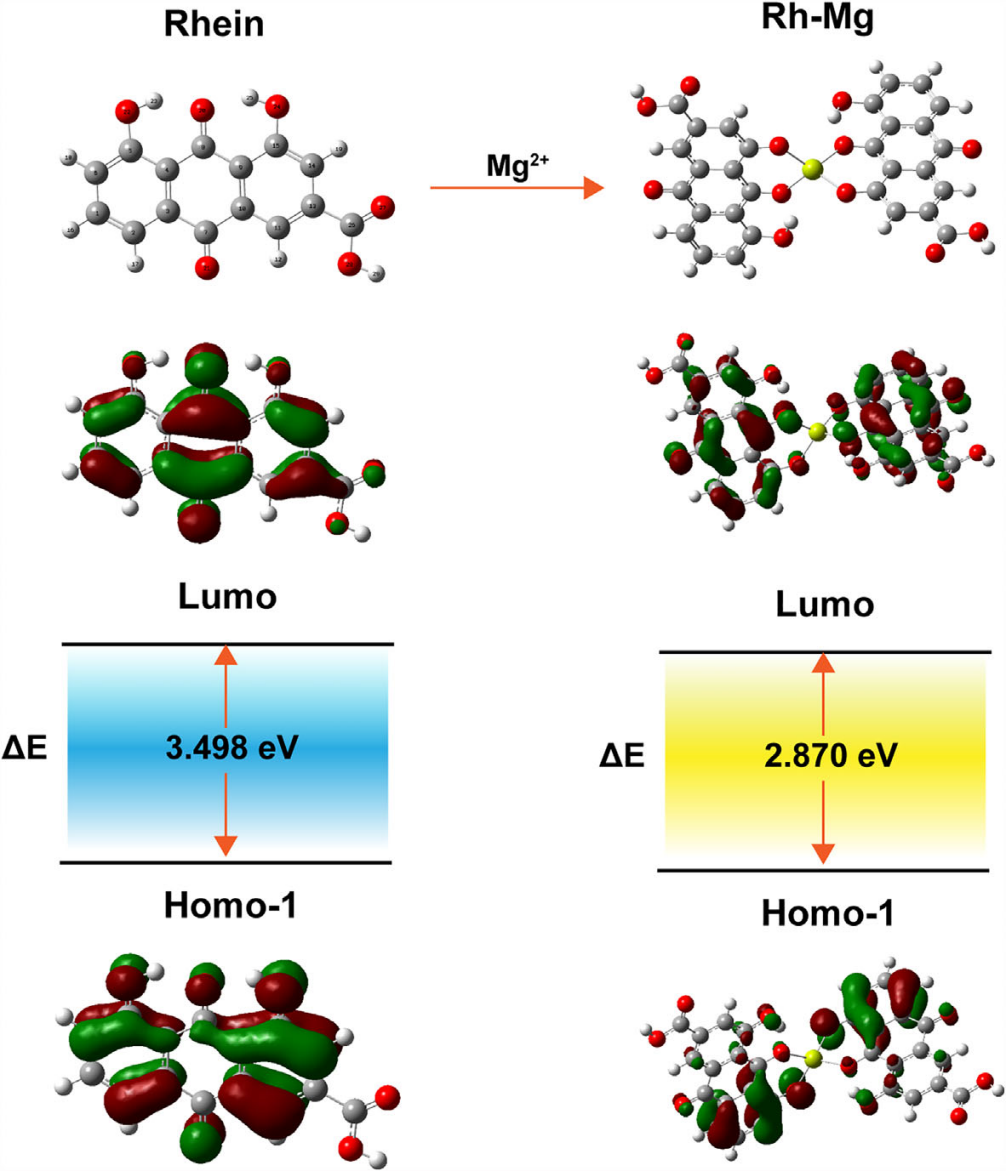
HOMO and LUMO orbitals of rhein and Rh-Mg.

The corresponding absorption and emission spectra of Rh-Mg-PVP@SiO_2_-TPP NPs were measured in water. The UV–vis absorption spectrum shows that the maximum absorption wavelength of Rh-Mg-PVP@SiO_2_-TPP NPs is 520 nm, similar to the spectrum of the Rh-Mg coordination polymer nanodots (**Figure 2A**). Compared with the maximum absorption wavelength of rhein at 430 nm, the spectrum of Rh-Mg-PVP@SiO_2_-TPP NPs has a red shift. To investigate the influence of magnesium on the UV–vis spectra of rhein, theoretical studies of electronic structures were carried out using the density functional theory (DFT) method (32). On the basis of optimizing the structure of ground state, the excited states of Rh-Mg and rhein in methanol were studied by the B3LYP/TD-DFT method. As shown in **Figure 3**, the energy gaps (*E*_gap_) of HOMO-1 and LUMO of rhein and Rh-Mg are 3.498 and 2.870 eV, respectively. Thus, forming a metal complex made a dramatic change in the energy levels of excited states, leading to the red shift in absorption spectra of Rh-Mg. When excited at 520 nm, Rh-Mg-PVP@SiO_2_-TPP NPs displayed strong emissions at 620 nm (**Figure 2B**). The results demonstrate that Rh-Mg-PVP@SiO_2_-TPP NPs exhibit large Stokes shifts, approximately 100 nm, which results in an efficient separation of the absorbance and emission maxima (**Figure 2C**). Next, we recorded the fluorescence quantum yields of Rh-Mg-PVP@SiO_2_-TPP NPs using the integrating sphere setting and got the value of 0.49 in water, which is a considerable value among many reported fluorescence probes (19, 26).

Probes used for biological imaging of live cells should have negligible interference with the proliferation of living cells at the concentration used for imaging. To evaluate their bioimaging potential, the cytotoxicity of Rh-Mg-PVP@SiO_2_-TPP NPs was measured by the CCK-8 assay after incubating Neuro-2a and BV2 cells. The concentration-dependent effects of Rh-Mg-PVP@SiO_2_-TPP NPs after 24 h of incubation are shown in **Supplementary Figure S4** (**Supporting Information**). After being treated with Rh-Mg-PVP@SiO_2_-TPP NP concentrations up to 200 μg ml^−1^, both Neuro-2a and BV2 cells showed more than 90% cell viability. This concentration was thus further used for live cell imaging. These results demonstrate that Rh-Mg-PVP@SiO_2_-TPP NPs have minimal cytotoxicity for cell imaging at a concentration of 200 μg ml^−1^.

**Figure 4:**
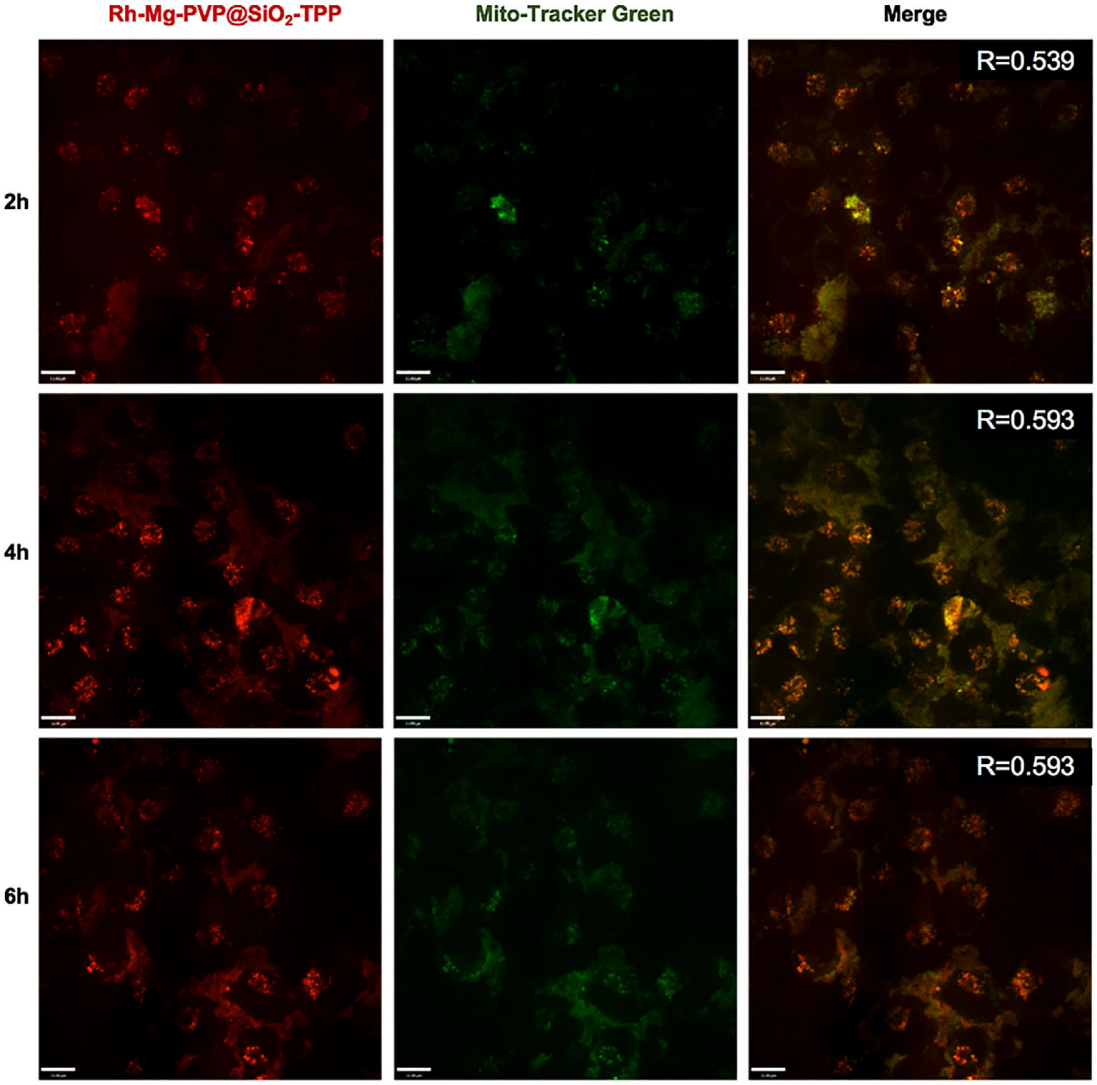
Co-localized images of live Neruo-2a cells incubated with 200 μg ml^−1^ Rh-Mg-PVP@SiO_2_-TPP in DMEM for different times at 37°C and then incubated with MitoTracker Green for 10 min at 37°C. Red channel for Rh-Mg-PVP@SiO_2_-TPP NPs (*λ*_ex_ = 561 nm), green channel for commercialized MitoTracker Green (*λ*_ex_ = 488 nm), co-localization of green and red channels. Scale bar represents 11 μm.

**Figure 5:**
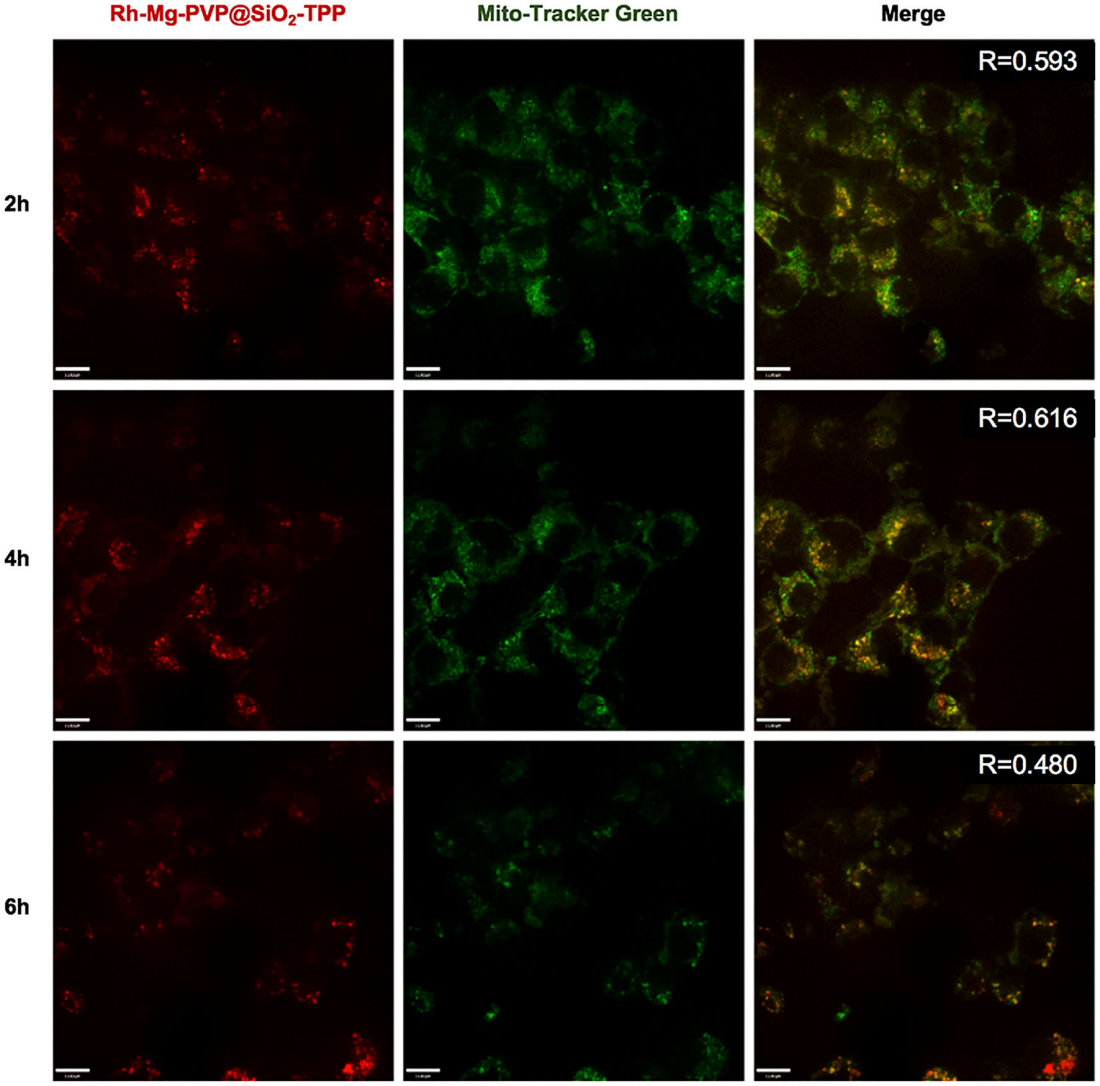
Co-localized images of live BV2 cells incubated with 200 μg ml^−1^ Rh-Mg-PVP@SiO_2_-TPP NPs for different times at 37°C and then incubated with MitoTracker Green for 10 min at 37°C. Red channel for Rh-Mg-PVP@SiO_2_-TPP NPs (*λ*_ex_ = 561 nm), green channel for commercialized MitoTracker Green (*λ*_ex_ = 488 nm), co-localization of green and red channels. Scale bar represents 11 μm.

To demonstrate the fluorescent imaging capability of Rh-Mg-PVP@SiO_2_-TPP NPs in cancer cells, we first performed cellular imaging of Neuro-2a cells under confocal laser microscopy. As shown in **Figure 4**, it was apparent that intracellular Rh-Mg-PVP@SiO_2_-TPP NPs emitted obvious bright red fluorescence and barely fluorescence in extracellular regions. This implied that Rh-Mg-PVP@SiO_2_-TPP NPs could enter into live cancer cells through simple incubation. In addition, a similar experiment was performed on BV2 microglial cells, where, similarly, a high uptake of Rh-Mg-PVP@SiO_2_-TPP NPs in live cell was observed (**Figure 5**). The co-staining experiment with a commercial probe is a common method to detect the selectivity of a new probe. Herein, MitoTracker Green (MTG), a commercial mitochondrial probe, was selected to detect the selectivity of Rh-Mg-PVP@SiO_2_-TPP NPs. The co-staining experiments of Rh-Mg-PVP@SiO_2_-TPP NPs and MTG have been performed on Neuro-2a and BV2 cells. The confocal fluorescent images of Neuro-2a cells incubated with Rh-Mg-PVP@SiO_2_-TPP NPs and MTG are shown in **Figure 4**. The red and green color fluorescent images were obtained after excited by 561 and 488 nm, respectively. The red fluorescence represented the distribution of Rh-Mg-PVP@SiO_2_-TPP NPs, and green represented MTG. As can be seen from the merged images, the red image and the green image were well merged after co-incubation of Rh-Mg-PVP@SiO_2_-TPP NPs and cell lines for 4 h. The mean co-localization coefficients of Rh-Mg-PVP@SiO_2_-TPP NPs and MTG from Neuro-2a cells and from BV2 cells were 0.59 and 0.61, respectively (**Figures 4**, **5**). These results demonstrated that Rh-Mg-PVP@SiO_2_-TPP NPs could stain mitochondria in live cells with high selectivity. In addition, we found that a portion of nanoprobes was also distributed in lysosomes (**Supplementary Figure S5**).

**Figure 6:**
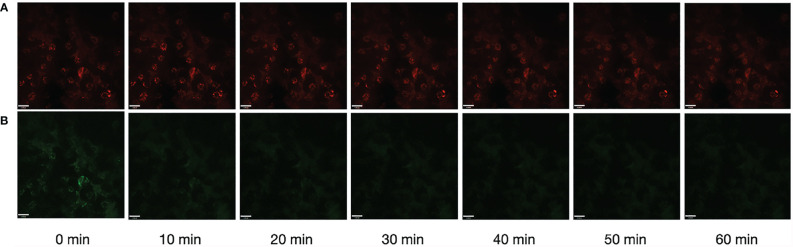
Photostability of fluorescence imaging of Neuro-2a cells with Rh-Mg-PVP@SiO_2_-TPP NPs **(A)** and MitoTracker Green **(B)**. Scale bar represents 11 μm.

Photostability is one of the most important parameters for fluorescent bioprobes. Here, to verify the work ability of Rh-Mg-PVP@SiO_2_-TPP NPs, the fluorescence imaging under long-time laser irradiation was investigated. After 1 h of laser irradiation, the fluorescence intensities of Rh-Mg-PVP@SiO_2_-TPP NPs in Neuro-2a cells remained at 97.1% (**Figure 6A**), whereas the fluorescence intensity of MitoTracker Green remained at 52.3% (**Figure 6B** and **Video S1**). The repeated experiment was performed in BV2 cells and similar results were obtained (**Supplementary Figure S6**). Thus, Rh-Mg-PVP@SiO_2_-TPP NPs could be believed as photostable, because it maintained more than 50% fluorescence intensities after 1 h of laser irradiation (**Figure 6A**). Therefore, the Rh-Mg-PVP@SiO_2_-TPP NPs show great potential for long-term live cell imaging.

In addition to cellular imaging, Rh-Mg-PVP@SiO_2_-TPP can also be used for *in vivo* imaging. Here, we selected the ICR mice as biological models, and the *in vivo* fluorescence imaging capability of Rh-Mg-PVP@SiO_2_-TPP NPs was investigated by MIIS small animal imaging system. After subcutaneous injection, compared with before injection, an obvious fluorescence signal was observed at the injection site (**Supplementary Figure S7** in **Supporting Information**). Moreover, the tissue distribution of Rh-Mg-PVP@SiO_2_-TPP NPs in mice was detected by *ex vivo* fluorescence imaging at different time points postintravenous injection. The fluorescence signal of organs was visible at 1 h after injection, gradually increased with time, and reached a maximum at 4 h (**Supplementary Figure S8** in **Supporting Information**). The overlays of fluorescence images and brightfield images of organs showed that the signal originated predominantly from the liver, kidney, and lung. These results suggest that Rh-Mg-PVP@SiO_2_-TPP NPs have excellent fluorescence properties, indicating their potential to be applied in bioimaging and diagnosis.

## Conclusion

In summary, a fluorescent nanoprobe with red fluorescence emission, strong light stability, large Stokes shift, and low cost was designed for mitochondrial imaging. The photophysical characteristics of Rh-Mg-PVP@SiO_2_-TPP NPs were systematically characterized *via* UV–vis absorption spectra, fluorescent emission spectra, and theoretical TD-DFT calculations. The Rh-Mg-PVP@SiO_2_-TPP NPs showed strong red emission and high quantum yield up to 0.49. In addition, Rh-Mg-PVP@SiO_2_-TPP NPs exhibited remarkable photostability and biocompatibility. After incubating Rh-Mg-PVP@SiO_2_-TPP NPs with the cancer cell line Neuro-2a and BV2 cells, Rh-Mg-PVP@SiO_2_-TPP NPs could specifically light up the mitochondria. Moreover, this nanoprobe can be used for *in/ex vivo* imaging. Our results provide inspiration for the design of nanostructures for mitochondrial imaging in cancer cells.

## Data Availability Statement

The original contributions presented in the study are included in the article/**Supplementary Material**. Further inquiries can be directed to the corresponding authors.

## Ethics Statement

The animal study was reviewed and approved by The Animal Care and Ethics Committee of Beijing University of Chinese Medicine.

## Author Contributions

XG designed the research. QJ and KD performed the experiments. YJ, YL, CH, and ZY analyzed the data. QJ and YW wrote the paper. XG and YW reviewed and edited the draft. All authors contributed to the article and approved the submitted version.

## Funding

This work was supported by the National Natural Science Foundation of China (81673562) and Fundamental Research Funds for the Central Universities of China (2020-JYB-ZDGG-033 and 2019-JYB-XJSJJ-006).

## Conflict of Interest

The authors declare that the research was conducted in the absence of any commercial or financial relationships that could be construed as a potential conflict of interest.

## Publisher’s Note

All claims expressed in this article are solely those of the authors and do not necessarily represent those of their affiliated organizations, or those of the publisher, the editors and the reviewers. Any product that may be evaluated in this article, or claim that may be made by its manufacturer, is not guaranteed or endorsed by the publisher.
