# Supplementary material for: Photostable Red-Emitting Fluorescent Rhein-Magnesium(Ⅱ) Coordination Polymer Nanodot-Based Nanostructures With a Large Stokes Shift for Imaging Mitochondria in Cancer Cell
Source: Front Oncol. 2021 Oct 25;11:758268. doi: 10.3389/fonc.2021.758268 (PMC8573231; doi:10.3389/fonc.2021.758268)
Supplement: Supplementary file 1 [file DataSheet_1.docx]

Supplementary Material

**Supplementary Figures**

**
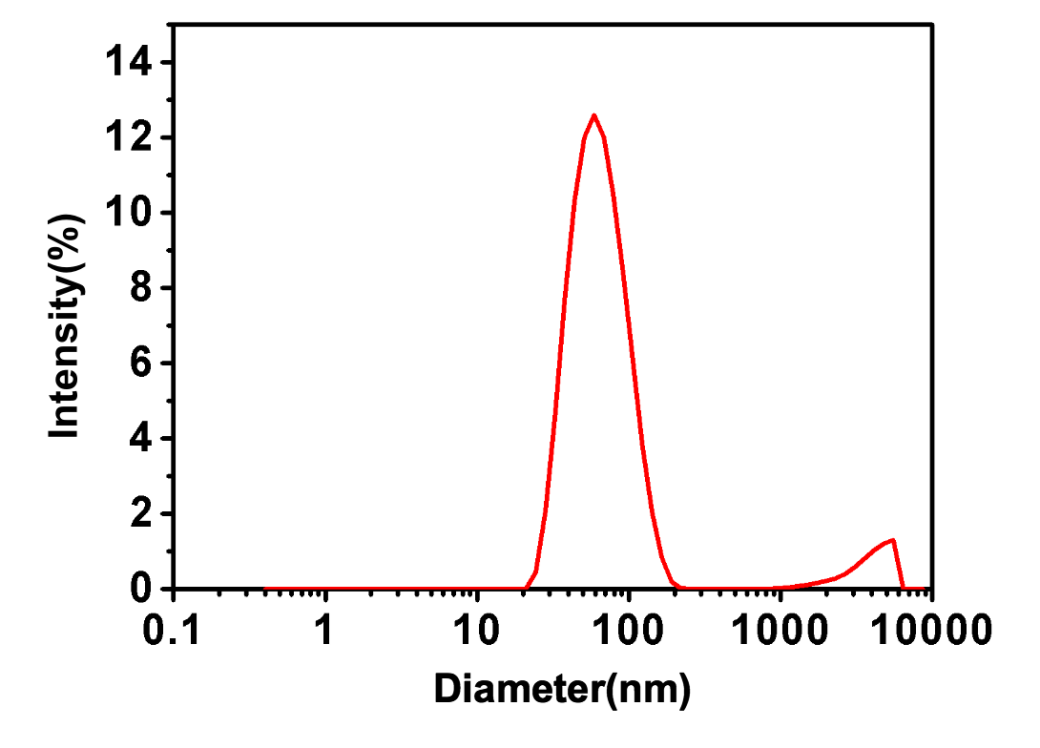
**

Supplementary Figure S1. The hydrodynamic size of Rh-Mg-PVP@SiO_2_.

_
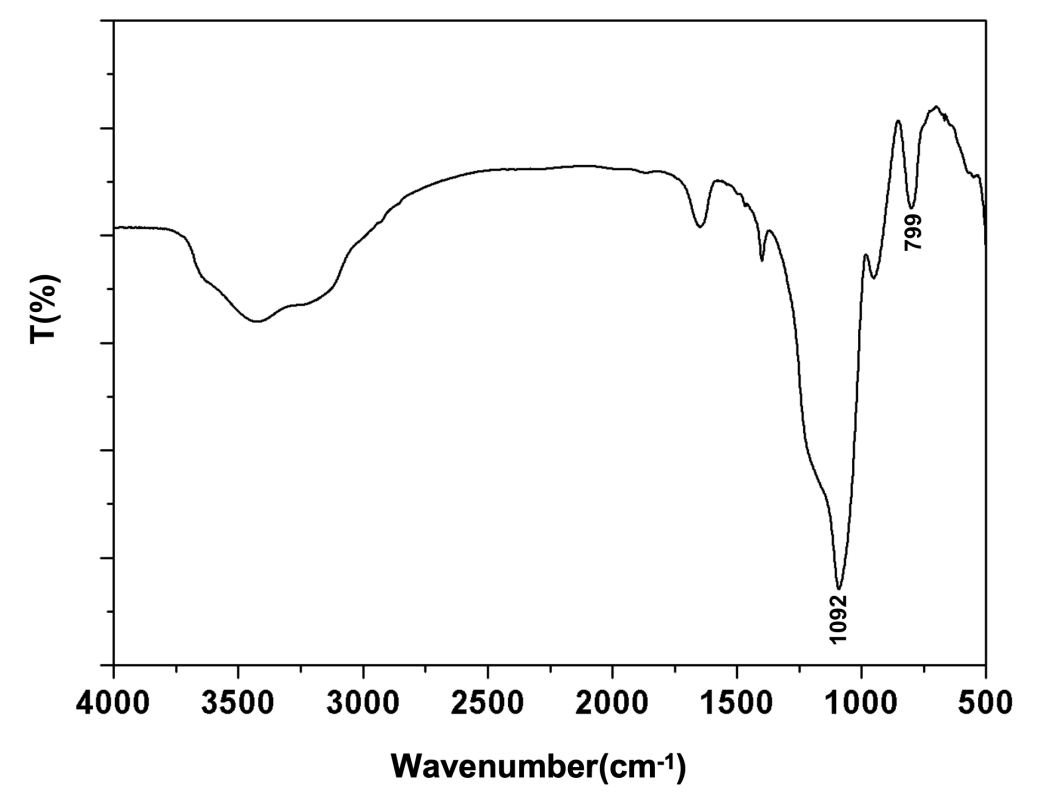
_

Supplementary Figure S2. FT-IR spectrum of Rh-Mg-PVP@SiO_2_.


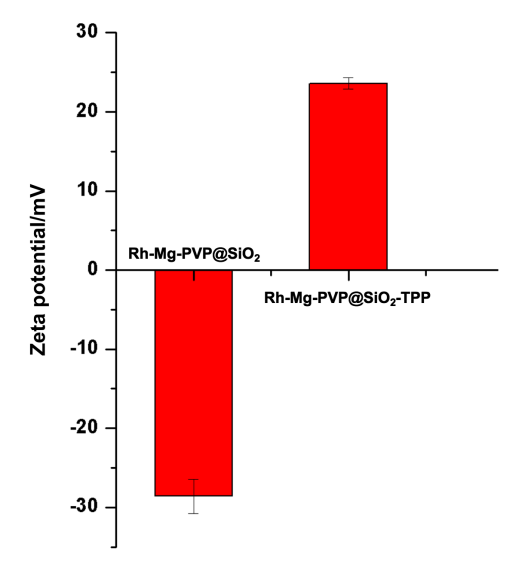


Supplementary Figure S3. *ζ* potentials of corresponding samples.


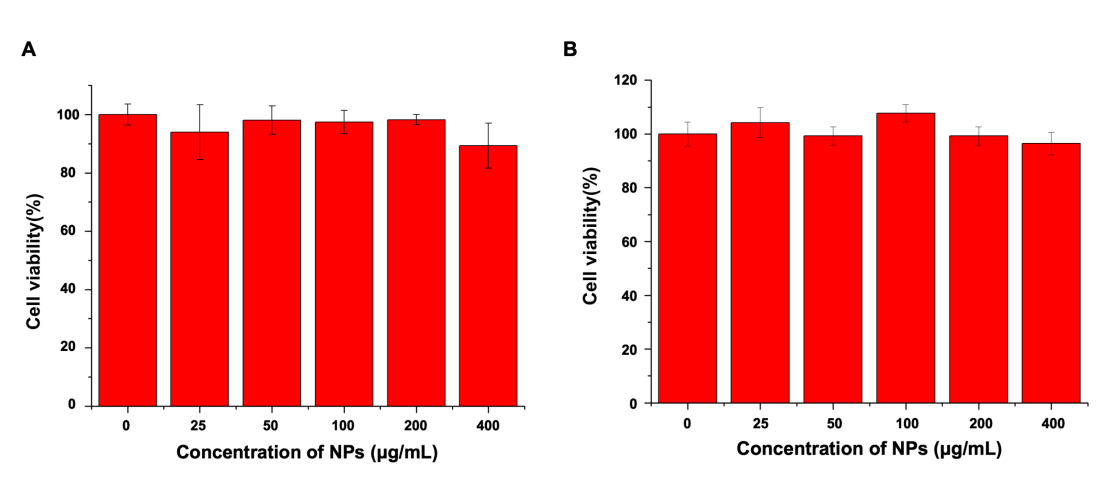


Supplementary Figure S4. CCK-8-based cytotoxicity assay of Rh-Mg-PVP@SiO_2_-TPP NPs using BV2 cells (A) and Neuro-2a cells (B). Cells are incubated with Rh-Mg-PVP@SiO_2_-TPP NPs for 24 h and then cell viability is calculated assuming 100 % viability for cells without any particles.

**
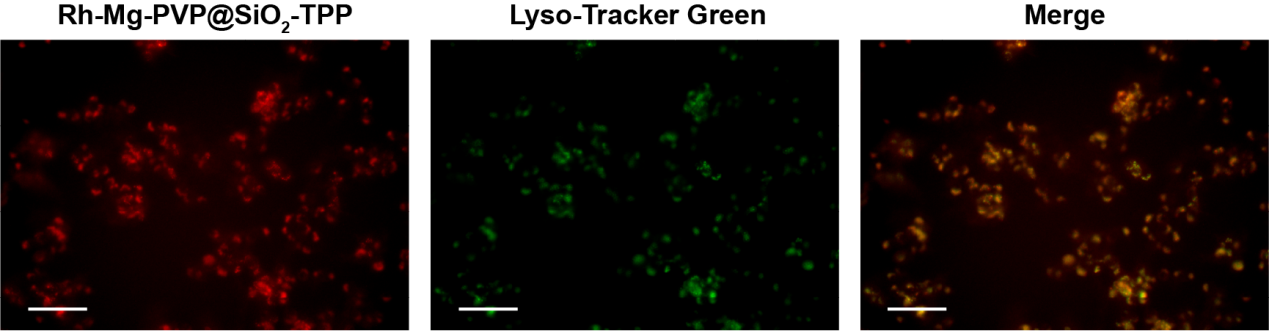
**

**Supplementary Figure S5.** Co-localized images of live BV2 cells incubated with 200 μg·mL^-1^ Rh-Mg-PVP@SiO_2_-TPP in DMEM for 8 h at 37℃ and then incubated with Lyso-tracker green for 10 min at 37℃. Red channel for Rh-Mg-PVP@SiO_2_-TPP NPs (λ_ex_ = 561 nm), green channel for commercialized Lyso-Tracker Green (λ_ex_ = 488 nm), co-localization of green and red channels. Scale bar represent 50 μm.

**
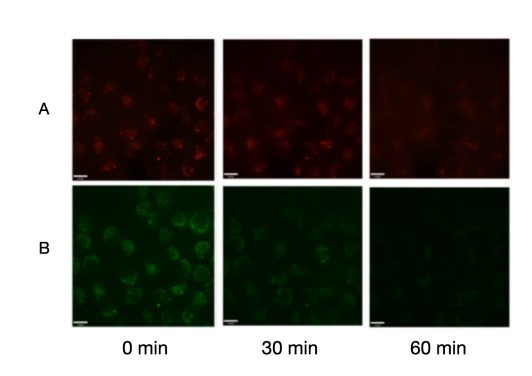
**

**Supplementary Figure S6.** Photostability of fluorescence imaging of BV2 cells with Rh-Mg-PVP@SiO_2_-TPP NPs (A) and Mito-Tracker Green (B). Scale bar represent 11 μm.


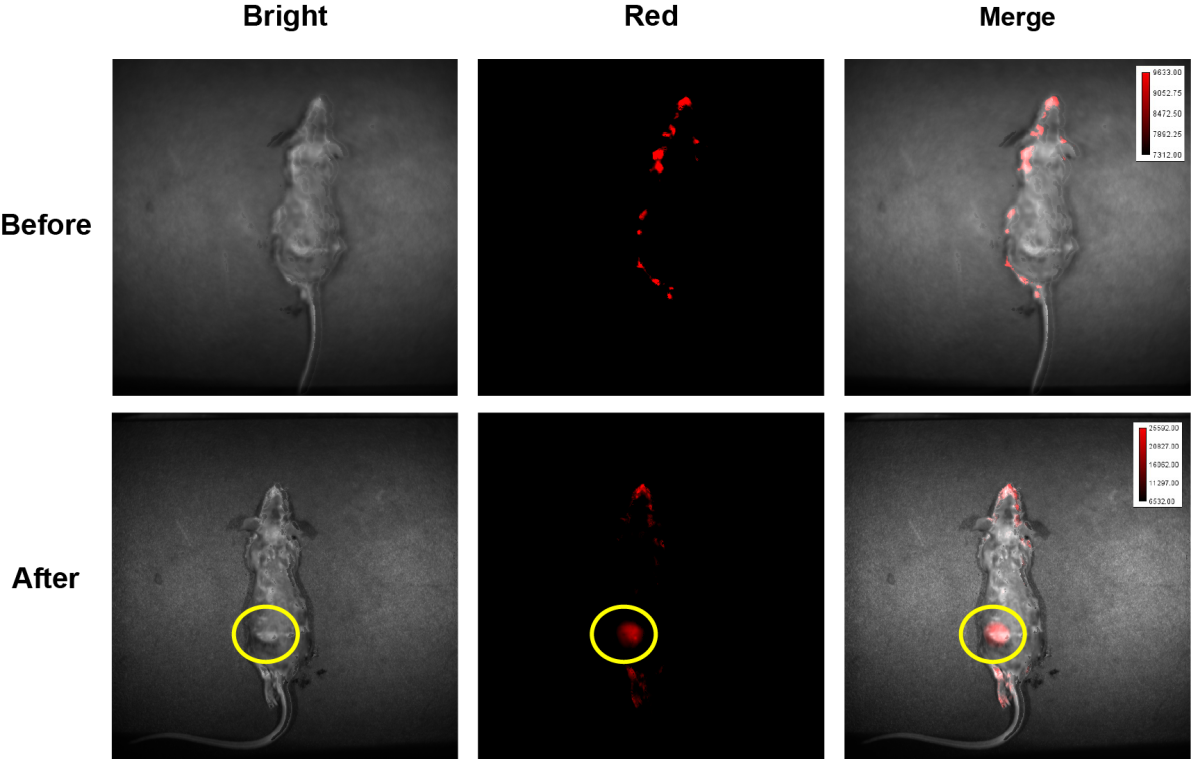


**Supplementary Figure S7.** *In vivo* fluorescence imaging of ICR mice before and after subcutaneous injection of Rh-Mg-PVP@SiO_2_-TPP NPs (6.848 mg/kg). The yellow circles point the injection sites of Rh-Mg-PVP@SiO_2_-TPP NPs.


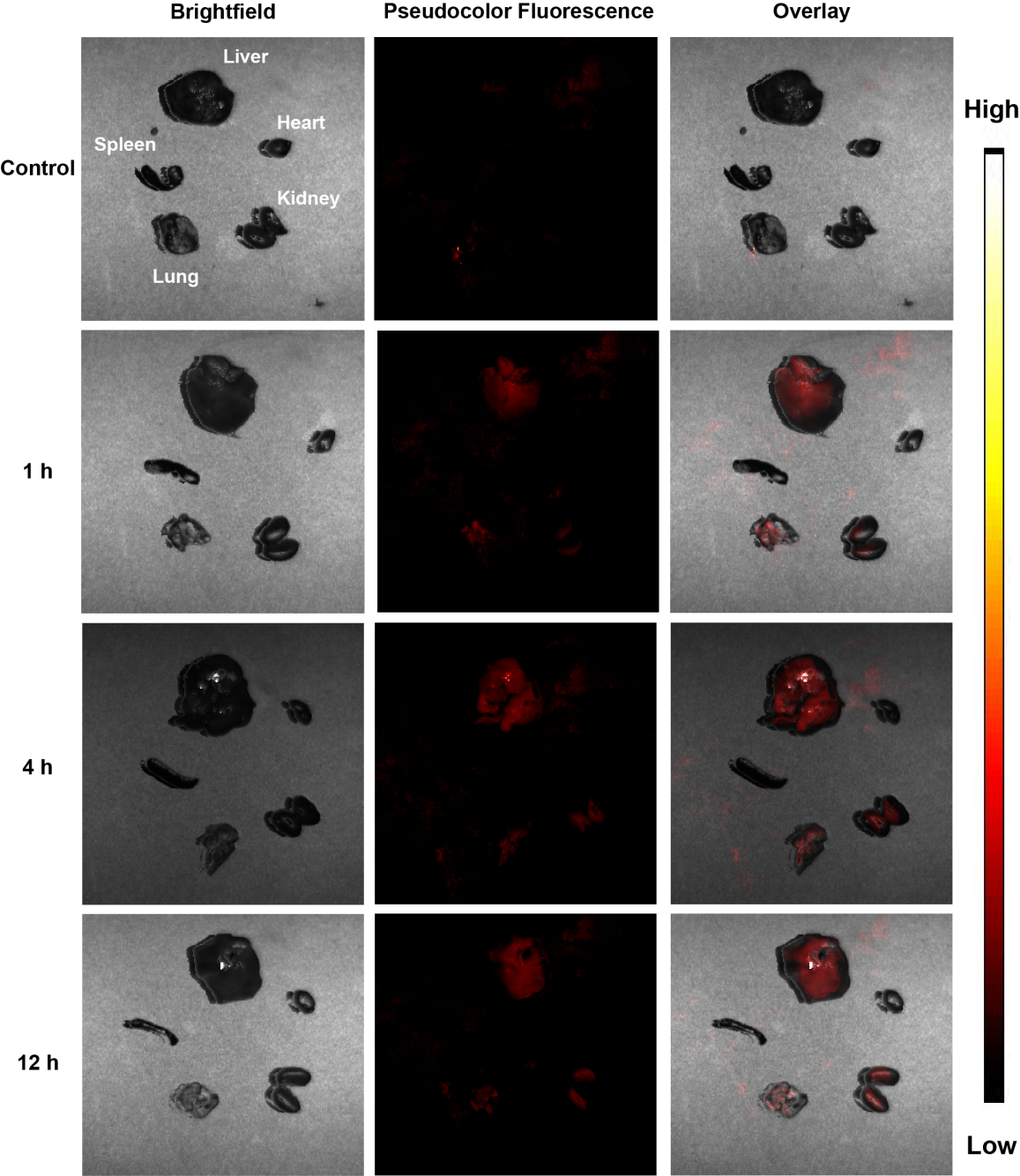


**Supplementary Figure S8.** Fluorescence images of the main internal organs harvested from ICR mice after intravenous injected with saline or Rh-Mg-PVP@SiO_2_-TPP NPs (6.848 mg/kg) at different time points (1, 4 and 12 h).

**Supplementary videos**
**Video S1.** Photostability of Rh-Mg-PVP@SiO_2_-TPP NPs was demonstrated using BV2 cells.
